# Tuning Surface Plasmonic Resonance and Surface Wettability of Au/CrN Films Using Nitrogen-Containing Gas

**DOI:** 10.3390/nano12152575

**Published:** 2022-07-27

**Authors:** Da-Hua Wei, Sheng-Kai Tong, Sheng-Chiang Chen, Yong-Han Hao, Ming-Ru Wu, Cheng-Jie Yang, Rong-Tan Huang, Ren-Jei Chung

**Affiliations:** 1 Institute of Manufacturing Technology, Department of Mechanical Engineering, National Taipei University of Technology (TAIPEI TECH), Taipei 10608, Taiwan; synder@ms6.hinet.net (S.-C.C.); a_25057389a@yahoo.com.tw (Y.-H.H.); fni50294@gmail.com (M.-R.W.); bruce.60705@gmail.com (C.-J.Y.); 2 Research and Development Department, CB-CERATIZIT Group, New Taipei City 24250, Taiwan; mmm.ntut@gmail.com; 3 Department of Optoelectronics and Materials Technology, National Taiwan Ocean University, Keelung 20224, Taiwan; 4 Department of Chemical Engineering and Biotechnology, National Taipei University of Technology (TAIPEI TECH), Taipei 10608, Taiwan

**Keywords:** CrN films, nitrogen-containing gas, surface wettability, crystal orientation transition, surface plasmonic resonance

## Abstract

The surface plasmonic resonance, surface wettability, and related mechanical nanohardness and of face-centered-cubic (fcc) chromium nitride (CrN) films have been successfully manipulated via the simple method of tuning nitrogen-containing gas with different nitrogen-to-argon ratios, varying from 3.5 (N35), to 4.0 (N40), to 4.5 (N45), which is directly proportional to argon. All of the obtained CrN films showed that the surface wettability was due to hydrophilicity. All of the characteristics were mainly confirmed and explained by using X-ray diffraction (XRD) patterns, including plan-view and cross-section SEM images, with calculations of the average grain size performed via histograms accompanied by different preferred grain orientations. In the present work, not only the surface plasmonic resonance, but also the surface wettability and the related mechanical nanohardness of CrN films were found to be tunable via a simple method of introducing adjustable nitrogen-reactive-containing gas during the deposition process, while the authors suggest that the crystal orientation transition from the (111) to the (200) crystalline plane changed significantly with the nitrogen-containing gas. So the transition of the preferred orientation of CrN’s cubic close-packed from (111) to (200) varied at this composite, caused and found by the nitrogen-containing gas, which can be tuned by the nitrogen-to-argon ratio. The surface plasmonic resonance and photoluminescence quenching effects were coupled photon and electron oscillations, which could be observed, and which existed at the interface between the CrN and Au metals in the designed heterostructures.

## 1. Introduction

Metallic alloy compounds composed of various elements have been attracting significant attentions in the research field of electronics, sensing, catalysis, biology, green energy, and surface plasmon resonance because of their excellent characteristics compared to bimetallic structures and their monometallic counterparts with corresponding electronic heterogeneity induced by elemental selection [1,2,3,4,5,6,7,8,9,10]. Surface plasmon resonance belongs to a coherent effect caused by electron oscillations that come from an interface between a metal and an agent. The excitation of coherent oscillations is highly dependent on the properties of the used metal, the structure and thickness of the metal, and the optical properties of the dielectric material [11,12,13]. Despite developments in nano- and bioscience and technology in the last 20 years, most of the sensing devices, detectors, and conductors are focused on nanostructures that are not easy to apply on a large scale. The surface coatings on transition nitride, semiconductor films, and related nanostructures are simple and the most effective method for observing plasmonic function-enhancement in the optical and electrical properties of underlying materials [14,15,16,17,18,19,20]. Nanostructures with plasmonics are absorbed in the photo-light near the metal semiconductor interfacial connection, which improves the strength of the coupled magneto-electric fields around plasmonic nanostructures. Because plasmonic nanostructures can concentrate the magneto-electro field that leads to the development of a direction of science and technology towards potential applications such as photovoltaics, photocatalysis, biosensors, biomedical optics, and spectroscopy and related imaging fields [21,22,23,24]. In addition, the coupling of plasmonics and excitons in metallic nanostructures combined with semiconducting materials has offered a direct solution to improving the plasmonic coupling and related sensitivity for a wide range of applications [25].

On the other hand, the surface wettability between the solid–liquid and liquid–liquid interfacial layers has received considerable attention due to its industrial utility and various potential applications, such as environmental cleanup and optical, optoelectronic, and photonic nanomaterials and related nanodevices [26,27,28,29,30]. Also, the surface wettability state is governed by the morphology, chemical composition, and surface free energy of solid surfaces. Among these, the surface wettability state is most closely related to the surface morphology which has been proposed in previous research studies from Cassie, Baxter, and Wenzel [31,32]. In the present work, the surface plasmonic resonance and wettability state of chromium nitride (CrN) films are demonstrated, with a special focus on the relationship between the surface morphology, water-contact angle, and surface free energy of the solid–liquid interface. The CrN films prepared using the physical vapor-deposition technique have been widely applied to protect the surface of the material, owing to their attractively outstanding properties in hardness, adhesion strength, ductility, wear, corrosion resistance, and high-temperature oxidation resistance [33,34,35,36,37,38,39,40]. Due to the aforementioned promising properties, we proposed that CrN films be applied to simultaneously satisfy both the surface-modification and mechanical requirements. However, not only the surface wettability state, but also the mechanical properties need to be evaluated, so that the research is much more comprehensive. Thus, we studied the nanoindentation technique, which enables the highly precise, dynamic measurement of the mechanical properties of the materials, such that it is useful to evaluate the mechanical properties of thin films in satisfaction of the requirements. Moreover, it has frequently been mentioned that the indentation depth should be less than one-tenth of the film’s thickness (the 1/10 rule) so as to ensure the minimum amount of noise from the substrates [41]. In this present work, face-centered-cubic (fcc) CrN films have been deposited by an unbalanced, direct-current, reactive magnetron sputtering system with nitrogen-containing gas with nitrogen-to-argon ratios varying from 35 to 45 sccm. Moreover, the surface free energy of the CrN films obviously decreased from 38.2 to 30.2 mJ/m^2^ in association with the varying nitrogen-containing gas contents ranging from 35 to 45 sccm. At the same time, the mechanical nanohardness was also increased from 1.52 to 3.02 GPa via the increased nitrogen-containing gas. In other words, our research revealed that all of the samples simultaneously demonstrated enhancements in mechanical nanohardness and surface free energy with increases in the amount of nitrogen gas. Thus, the plan-view and cross-sectional micrographic images indicate that the grain size and the diameter of the columnar structure decreased slightly with increases in the amount of nitrogen gas. All of the samples also exhibited clear, dense, and highly uniform characteristics.

This work also demonstrated that tunable surface wettability and mechanical nanohardness can be easily controlled via the simple method of tuning the nitrogen-containing gas. Hence, such multifunctional CrN films can be the promising candidates for various potential applications, offering outstanding surface wetting characteristics in the solid–liquid interface and control over the mechanical properties. There exist only a few reports dealing with metal ions/nanoclusters embedded in transition nitride polycrystalline films [42,43]. We simultaneously aimed to investigate the coupling of surface plasmons and excitons via Au/CrN structures, as the coupling strengths of the metals are different. The mechanism responsible for the enhancement of surface plasmonic resonance has been confirmed.

## 2. Experiments and Composite Film Structures

In the present work, CrN films were fabricated by an unbalanced, direct-current, reactive magnetron sputtering system. In addition, the CrN films were directly deposited onto Corning 1737 glass substrates for 120 min with a working power of 50 watts and a working temperature of 400 °C. We introduced argon (10 sccm) and nitrogen (35, 40, and 45 sccm) gases as carrier gases in different nitrogen-to-argon ratios: 3.5, 4.0, and 4.5, respectively, which was directly proportional to the amount of argon present during the deposition process. All of the substrates (10 × 10 mm^2^) were set parallel to the commercial chromium target with a 4N purity rating (99.99%), which were 2 inches and 3 mm in diameter and thickness, respectively. Sequentially, the glass substrates were treated with acetone, ethanol, and isopropanol to remove any organic contaminants, and then they were finally rinsed in deionized water via an ultrasonic cleaner for over 10 min between each cleaning step. After that, all of the substrates were dried with compressed air before being loaded into the vacuum chamber. In addition, the base pressure of the high-vacuum chamber was lowered to 5 × 10^−7^ torr via a mechanical backing pump and a molecular turbo pump for over 8 h. Before the deposition process, the pure argon and nitrogen (99.99%, 4N) gases were introduced into the vacuum chamber from the base pressure (5 × 10^−7^ torr) to the working pressure (1 × 10^−2^ torr) without introducing oxygen gas at any point during the sputtering process.

The crystal orientation and microstructure of the CrN films were employed by a θ-2θ scan using X-ray diffraction (XRD, PANalytical, Almelo, The Netherlands) with Cu Kα radiation (λ = 1.54 Å) of 2θ = 20–60°. Examinations of the surface morphology and cross-section view of the CrN films were performed by field emission scanning electron microscopy (FE-SEM, Dresden, Germany) and (Phenom XL G2, Thermo Scientific, MA, USA). The surface wettability of the CrN films was observed via the contact-angle (CA, Rame-Hart 100 goniometer, Capovani Brothers Inc., NY, USA) measurement of water droplets (10 μL) at the interface between the air and the solid surface of each sample. The tolerance of the contact-angle measurement was slightly influenced by the image quality, using a charge-coupled device (CCD) to capture the images of the water droplets, and the finishing curve of the fitting function was obtained automatically using CA software, and this was estimated to be about ±1 degree. On the other hand, the corresponding surface free energy (SFE) of each measurement was also calculated at the same time. The nanoindentation measurement was employed to determine the mechanical nanohardness (Hysitron TI 980 TriboIndenter, Bruker, Billerica, MA, USA) of the CrN films with the typical load versus the displacement curves, and each sample was measured three times to ensure accurate hardness values. In this work, the as-prepared CrN films were subjected to nitrogen-to-argon ratios that varied from 3.5, to 4.0, to 4.5 without any post-treatment, and these ratios were denoted as N35, N40, and N45, respectively.

## 3. Results and Discussion

**Figure 1:**
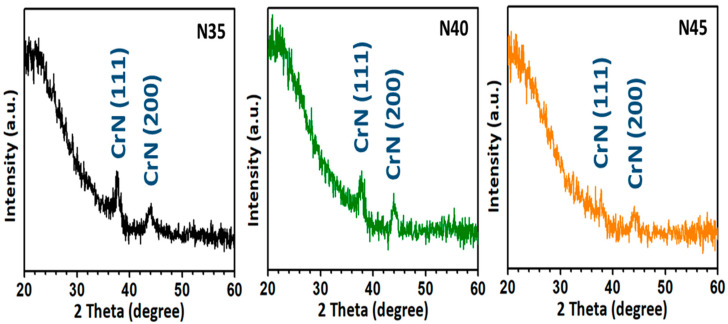
XRD patterns for all CrN films with nitrogen-to-argon ratios varying from 3.5 (N35), to 4.0 (N40), to 4.5 (N45), respectively.

The microstructure and crystal orientation of the CrN films deposited onto the glass substrates, with nitrogen-to-argon ratios varying from 3.5, to 4.0, to 4.5, were obtained using XRD with a θ–2θ scan, as shown in Figure 1. The XRD patterns confirmed the crystalline structure of the face-centered-cubic CrN films with an Fm-3m (225) space group, and there was no impurity phase that could be observed in any of the XRD patterns. In addition, it could be clearly observed that two diffraction peaks were located at around 2θ = 37° and 43° in the N35 and N40 samples, which can be indexed to the CrN (111) and (200) facets, respectively. On the other hand, both diffraction peak intensities of the CrN (111) and (200) facets in sample N45 were dramatically decreased with increases in the density of the nitrogen-containing gas from 35 to 45 sccm. This phenomenon could be attributed to the decreased mobility of the Cr adatoms in the reactive atmosphere with a saturated nitrogen ambience during the deposition process. Thus, redundant nitrogen segregated to the surface and grain boundaries, which extremely reduced the mobility of the Cr surface and grain boundaries, limiting the grain-coarsening during coalescence and film growth [44,45]. Similar results have been reported in previous research [46,47,48,49,50].

**Figure 2:**
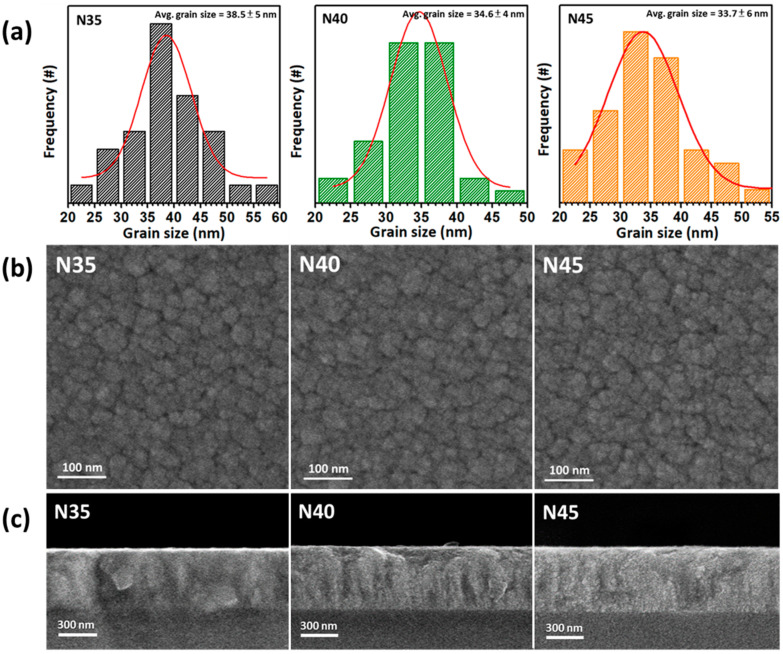
The plan-view and cross-sectional FE-SEM images with calculated average grain sizes for all CrN films with different nitrogen-to-argon ratios: (**a**) the calculated average grain sizes performed via histograms; (**b**) plan-view images; and (**c**) cross-sectional images.

The plan-view and cross-sectional SEM images, with calculated average grain-sizing performed via histograms of the face-centered-cubic CrN films deposited onto the glass substrates with nitrogen-to-argon ratios varying from 3.5 (N35) to 4.5 (N45) are shown in Figure 2, respectively. As shown in Figure 2b, the plan-view SEM images demonstrate a granular structure with highly uniform and dense characteristics that demonstrate roughly the same type of morphology in samples N35, N40, and N45. Figure 2a reveals that the corresponding histograms for the calculated average grain size and distribution of the CrN films deposited onto glass substrates with nitrogen-to-argon ratios varying from 3.5, 4, to 4.5, respectively. The values of the average grain sizes corresponding to the samples N35, N40, and N45 were 38.5 ± 5 nm, 34.6 ± 4 nm, and 33.7 ± 6 nm with minuscule deviation, respectively. The above results indicate that the slight variation in average grain size obtained with varying nitrogen-to-argon ratios, from 3.5, to 4, to 4.5. They also reveal the decreasing average grain size with increases in the mass flow of partially introduced nitrogen gas during the deposition process from 35 to 45 sccm. This phenomenon could be also attributed to the reduced mobility of Cr adatoms under the saturated ambience during the deposition process, limiting the grain coarsening and leading to the secondary nucleation. The cross-sectional SEM images of the CrN films deposited onto the glass substrates with different nitrogen-to-argon ratios are as shown in Figure 2c. The cross-sectional SEM images of samples N35, N40, and N45 show a columnar structure and very smooth surface characteristics, as shown in Figure 2c. This demonstrates that the thicknesses of the film samples were around 500 nm and had consistent columnar structures. Even though the samples developed columnar structures, there still exist slight difference among their crystal orientations. The cross-sectional image of sample N35 shows a columnar structure accompanied by coarse grains, and sample N40 demonstrates a slender columnar structure accompanied by fine grains as well. On the other hand, the cross-sectional image of sample N45 exhibits highly uniform and dense characteristics with a skinny columnar structure without any coarse grains.

**Figure 3:**
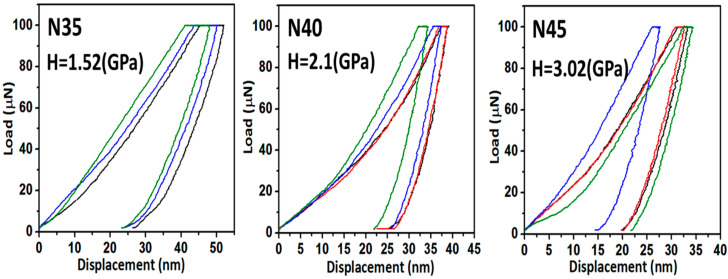
The typical load versus the displacement curves for the CrN films and the average nanohardness values due to different nitrogen-to-argon ratios.

The typical load versus the displacement curves for each CrN film were measured three times and deposited onto the glass substrates with different densities of nitrogen-containing gas, varying from 3.5 to 4.5, as shown in Figure 3. For the mechanical characteristic measurements, nanoindentations were conducted on all of samples using a Berkovich diamond indenter. After the indenter landed on a sample, the load was increased at a predetermined rate (10 μN/min) to the desired maximum load of 100 μN, and then it was decreased at the same rate (10 μN/min) until it reached zero. Then, the load was plotted against the displacement of the indenter for each loading and unloading cycle. Moreover, it was necessary to confirm that the maximum indentation depth for the CrN films was less than approximately 50 nm. This depth penetration is small enough to avoid the size indentation effect, which is considered to be 10% of the film’s thickness. Figure 3 shows maximum indentation depths of around 50, 40, and 35 nm for samples N35, N40, and N45, respectively. Thus, the CrN films deposited under different densities of nitrogen-containing gas, varying from 3.5, to 4, to 4.5, exhibited average nanohardness values of 1.52, 2.1, and 3.02 GPa for samples N35, N40, and N45, respectively. It is noteworthy that sample N45 had a higher nanohardness of 3.02 GPa as compared to samples N35 and N40, and this was attributed to multiple reasons. One of them, according to the results of SEM plan-view images and corresponding histograms for the calculated average grain size with the distribution of CrN films, is shown in Figure 2a,b. The average grain size of all of the samples decreased with the increasing density of nitrogen-containing gas, varying from 3.5 to 4.5. Thus, in general metallic materials, yield stress (σ_y_) is related to grain size (d) through the Hall–Petch equation:

σ_y_ = σ_0_ + kd^−1/2^ (1)

where σ_0_ is the friction stress in the absence of grain boundaries, and k is a positive constant of yielding associated with the stress required to extend dislocation activity into neighboring, unyielded grains [51,52]. Obviously, this reveals the inversely proportional relationship that increased yield stress has with decreasing grain size. In brief, this phenomenon could be explained as grain-boundary strengthening. The grain boundaries play an important role as pinning points that limit further dislocation propagation, which also prevents dislocations from moving in a continuous slip plane [53]. In other words, the decrease in grain size will improve mechanical characteristics, such as hardness and yield stress, which is the reason why so much effort is taken to obtain ultrafine and even nano-grained materials. Second, because of the increased density of the nitrogen-containing gas, varying from 3.5 to 4.5, the sample demonstrated a decreased diffraction intensity ratio with CrN_(111)/(200)_. However, the tendency was similar between the nanohardness and XRD patterns, but according to the literature, an explanation of the relationship between nanohardness and crystal orientation, having the exact same case, is still lacking. Hence, despite of the many results, we still can organize similar cases that can be compared with each other. The CrN films were prepared by an ion-source-enhanced, middle-frequency, magnetron sputtering system with an ion source that varied between 600 and 1000 V. In addition, it showed the phase transition from the CrN(111) facet’s preferred orientation to the CrN(200) facet plane, not only exhibiting the same tendency as the decreased diffraction intensity ratio CrN_(111)/(200)_ with an increase in the ion source, but it also demonstrated the inverse relationship results regarding nanohardness. It is worth noting that the CrN(200) films dominated and had the highest hardness. Furthermore, the deposition of monolithic CrN coatings with direct-current magnetron sputtering (DCMS) and high-power, impulse magnetron sputtering (HIPIMS) techniques, as a function of the substrate rotation (zero-, one-, two- and three-fold substrate rotations), were performed and compared. Additionally, the XRD patterns and mechanical hardness of the CrN coatings which were prepared using the DCMS mode showed a relatively high mechanical hardness with a lower diffraction intensity ratio of CrN_(111)/(200)_. On the other hand, the mechanical hardness of the CrN coatings fabricated using the HIPIMS mode were similar when compared with each other, with only minuscule deviations, and this similar tendency was also shown by the XRD patterns [48]. The above results reveal the relatively low diffraction intensity ratio of CrN_(111)/(200)_, or it could be dominated by the CrN(200) facet, which shows a relatively high mechanical hardness and similar results and tendencies consistent with our present work. Thus, it can be concluded that sample N45 had a nanohardness, 3.02 GPa, than did samples N35 and N40, and this can be attributed to the coherent grain-boundary strengthening accompanied by the dominated CrN(200) lattice. It has been reported that coherent grain boundaries have very little energy, and they are very stable, with enhanced hardness and toughness. The hardness and toughness of the CrN coatings were improved simultaneously via the distortions that were formed during the transition from the (111) and (200) planes. This kind of grain boundary is identified as a coherent grain boundary, and it has a large number of multiple dislocations and 2–3 times the thickness of a conventional grain boundary [54,55]. When extended screw dislocation intersects a coherent grain boundary under an applied stress, the dislocation can directly traverse the coherent grain boundary, with the incoming screw being absorbed into the grain boundary and then desorbed through the process of cross-slip [56].

**Figure 4:**
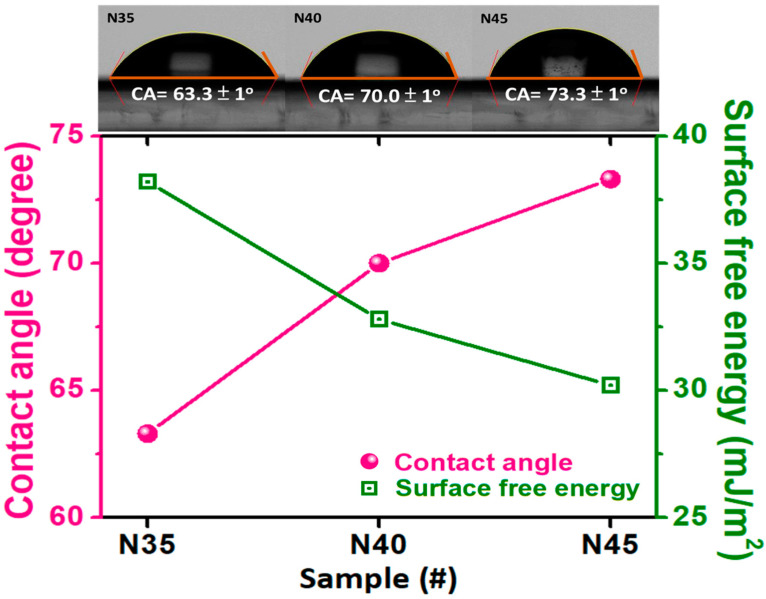
Water-contact-angle (WCA) images and surface free energy for all CrN films with different nitrogen-to-argon ratios.

In order to determine the wetting behavior of the CrN films, we measured the corresponding water-contact angles (WCAs) and surface wettability of the CrN films deposited onto the glass substrates with different densities of nitrogen-containing gas, varying from 3.5 to 4.5, as shown in Figure 4. The values of the water-contact angles for samples N35, N40, and N45 were 63.3 ± 1°, 70.0 ± 1°, and 73.3 ± 1°, respectively. Also, all of the samples demonstrate values of contact angles less than 90°, which could be classified as hydrophilic wettability. In addition, the values of the water-contact angles were increased with the increasing the density of the reactive gas. This phenomenon could be attributed to an air pocket forming and existing between the grains and against the grain boundaries. In other words, air pockets play an important role in supporting the water droplets in the case of the penetration, and they lead to higher water-contact angles [57,58,59,60,61], hence the similar, corresponding tendency of the SEM images with calculated average grain sizes performed via histograms, as shown in Figure 2a. Moreover, the histograms of the calculated average grain sizes show the same tendency, with the gradually increasing values of the water-contact angles.

On the other hand, not only the water-contact angles, but also the surface free energy of the corresponding CrN films with different densities of nitrogen-containing gas, varying from 3.5 to 4.5, are demonstrated in Figure 4**.** In addition, the commonly employed Fowkes–Girifalco–Good (FGG) theory was used to determine the surface free energy of the nonpolar solid–liquid interface [62,63,64,65,66,67]. The Fowkes equation is described as follows:

γ_sl_ = γ_s_ + γ_l_ − 2(γ_s_^d^γ_l_^d^)^0.5^ (2)

where γ_sl_, γ_s_, and γ_l_ are the surface free energy of the solid–liquid interface, a solid surface, and non-polar liquid surface, respectively. Furthermore, γ_l_^d^ and γ_s_^d^ are the dispersion of the surface tension for the liquid and solid surfaces, respectively. Additionally, the Fowkes equation (FGG) can be combined with Young’s equation, as the Girifalco–Good–Fowkes–Young (GGFY) equation, due to the fact that γ_l_^d^ and γ_s_^d^ can be equal to γ_l_ and γ_s_, respectively, using deionized water (a nonpolar liquid), with γ_l_ = 72.8 mJ/m^2^ during the measurements, and it can be simplified as in the following formula:

γ_s_ = 0.25 γ_l_ (1 + cosθ)^2^ (3)

where θ is the contact angle between the solid–liquid surface measured via water-contact-angle measurements [68]. In the present work, we simultaneously measured the surface free energy of the CrN films with different densities of nitrogen-containing gas, varying from 3.5 to 4.5, by using the GGFY equation, as shown in Figure 4. The surface free energy of samples N35, N40, and N45 were 38.2, 32.8, and 30.2 mJ/m^2^, respectively. The above results show that the surface free energy was sequentially decreasing with the increasing amount of nitrogen-containing gas, which ranged from a 35 to a 45 sccm mass flow-rate. Also, the corresponding tendency could obviously be varied, such that the value of surface free energy decreased by 26.5% for samples N35 and N45. Besides, the results of the surface free energy and the calculated average grain size are strongly related, owing to the trapped air pockets existing between the grains and against the grain boundaries. Our present results indicate that the density of nitrogen-containing gas plays an important role in simultaneously tuning and changing the wettability, surface free energy, and nanohardness of the designed CrN films. This work not only demonstrates a simple method of surface modification that changes the surface wettability and mechanical properties by introducing different densities of nitrogen-containing gas, but it also paves the way for other potential applications, such as self-cleaning, anti-dust/fog and anti-stick surfaces. In addition, this work simultaneously feeds the appetite for good mechanical hardness and anti-scratch properties, and the films can even be used to deposit coatings on the surfaces of molds.

**Figure 5:**
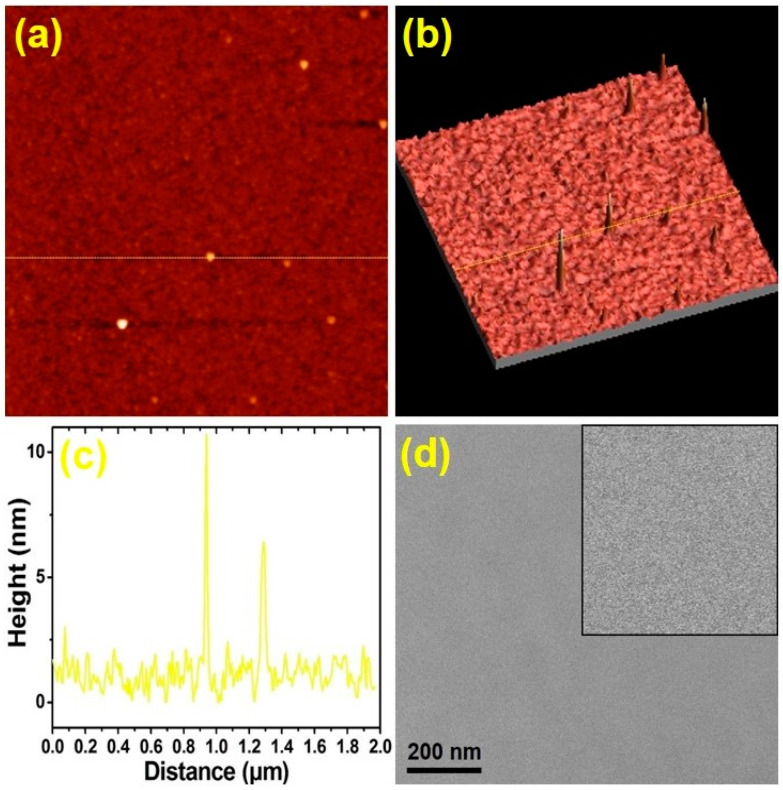
AFM morphology of the Au/CrN films: (**a**) 2D topical image; (**b**) 3D image of (**a**); and (**c**) a line-scan analysis of the Au/CrN films corresponding to (**a**,**b**). The scan area is 2 × 2 μm^2^. (**d**) a plan-view of the SEM image; the inset is an enlarged image of the area.

In order to study the plasmonic effect, a 10 nm thick Au nanolayer was selected to deposit onto the CrN (N40) films, which was denoted as Au/CrN. The Au film existed in a random cluster structure with an island mode growing on it, as observed by a typical, two-dimensional (2D) AFM micrograph, as shown in Figure 5a. This aspect of island growth is also supported by the three-dimensional (3D) AFM micrograph, as shown in Figure 5b. From the AFM line-scan, as shown in Figure 5c, the size and height of the irregular Au cluster structures were observed. The plan-view SEM image of the Au/CrN40 films showed highly uniform characteristics, and generally displayed a flat surface morphology, as shown in Figure 5d. An Au nanolayer, shown in Figure 5, supports the formation of Au-coated CrN films, which can be indexed to Au/CrN bilayer nanostructures in a cubic phase. The above morphological results analysis supports the configuration of the Au/CrN films, which is consistent with the observation made based on topical AFM and SEM images, as shown in Figure 5.

**Figure 6:**
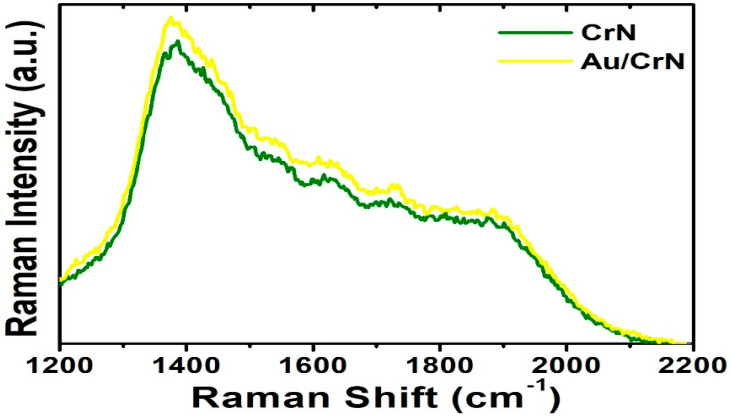
Raman spectra measured at room temperature for CrN films, with and without Au nanostructures, respectively.

On the other hand, surface plasmon resonances (SPRs) are coupled photon and electron oscillations that can exist at the interface between the CrN and Au metals in our designed heterostructures. The nanostructured metals, which provide the surface plasmon with collective oscillations of free electrons, can concentrate electromagnetic (EM) fields to a small fraction of a wavelength while enhancing local field strengths [69]. The local EM field experienced by analyte molecules on the metal surface in nanoscale is dramatically enhanced, producing strongly enhanced Raman intensity [70]. In the CrN films capped with the Au nanostructures that could induce localized field around its surface, there was a coupling effect that exists in such heterostructures, exhibiting the SPRs. A surface-enhanced Raman scattering (SERS) effect was observed in the Au/CrN heterostructures, as shown in Figure 6. Au nanostructures could offer an effective path to more photonic scattering from incident light. In order to enhance absorption of light in molecules and increase Raman scattering intensities via surface plasmon, it has been claimed that they are excited by the interaction between light and metal surfaces [71,72]. Since the appearances of the surface plasmon resonance bands in this region are characteristic of the surface plasmon resonance of Au nanostructures, this is an indication of the formation of Au nanostructures on the CrN layer. This is in agreement with the morphological analyses measured by AFM (Figure 5), where the formation of Au nanostructures has been confirmed.

**Figure 7:**
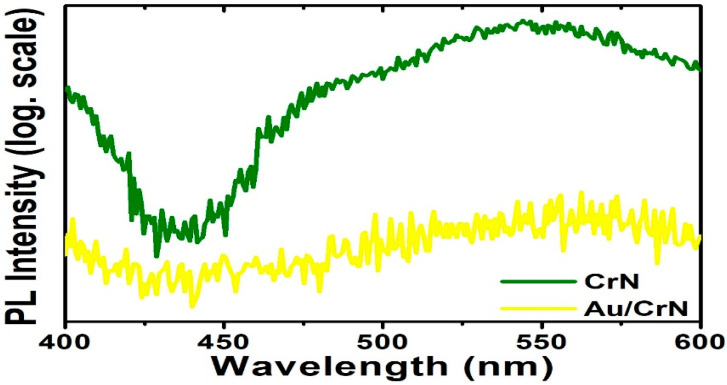
Photoluminescence (PL) spectra measured at room temperature for CrN films, with and without Au nanostructures, respectively.

The photoluminescence (PL) quenching in the Au/CrN heterostructures may also be due to the coupling of the light emission with the localized surface plasmon (LSP) resonance of the Au nanostructures, as shown in Figure 7. On the other hand, the metallic LSP can trigger both plasmonic excitation and interband excitation [73,74]. The Au nanostructures associated with LSP could also lead to the generation of the photoelectron process, owing to its absorption cross-section and localized optical intensity. Therefore, our work presents the functional Au/CrN heterostructures that could be developed in SERS detection and further applied in combination with hybrid devices, such as biosensors, with potential application in sub-marine UV photodetector designs [75,76]. The potential candidates, such as transition metal nitrides, have excellent electrical conductivity and superior cycling stability that could be used in supercapacitor electrode materials. So, CrN thin films also have great potential for application in symmetric supercapacitors and other energy storage systems [77,78,79,80].

## 4. Conclusions

Face-centered-cubic CrN films have been successfully fabricated onto Corning 1737 glass substrates via an unbalanced, direct-current, reactive magnetron sputtering system with nitrogen-to-argon ratios varying from 3.5, to 4.0, to 4.5, respectively. The surface wettability and mechanical nanohardness of the CrN films were enhanced via the transition of the preferred orientation of the CrN from (111) to (200) and by decreasing the average grain size. On the other hand, the surface plasmonic resonance are coupled photon and electron oscillations that can exist at the interface between CrN and Au metals in our designed heterostructures. The enhanced Raman intensity and photoluminescence quenching in the Au/CrN heterostructures could be observed due to the coupling of the light emission with the localized surface plasmon resonance of the Au nanostructures, which could be used for detection and energy-storage applications in the future.

## Data Availability

Data are contained within the article.
